# Increased plasma DR‐70 (fibrinogen‐fibrin degradation products) concentrations as a diagnostic biomarker in dogs with neoplasms

**DOI:** 10.1111/jvim.16898

**Published:** 2023-10-14

**Authors:** Chiao‐Hsu Ke, Ka‐Mei Sio, Chun‐Hung Wu, Yuan‐Yuan Xia, Jih‐Jong Lee, Chin‐Hao Hu, Cheng‐Chi Liu, Chueh‐Ling Lu, Chiao‐Lei Cheng, Keng‐Hsuan Lin, Hirotaka Tomiyasu, Yu‐Shan Wang, Chen‐Si Lin

**Affiliations:** ^1^ Department of Veterinary Medicine, School of Veterinary Medicine National Taiwan University Taipei 10617 Taiwan, ROC; ^2^ Wellcarevet Animal Hospital Taipei 11460 Taiwan, ROC; ^3^ Graduate Institute of Veterinary Clinical Science, School of Veterinary Medicine National Taiwan University Taipei 10617 Taiwan, ROC; ^4^ Animal Cancer Center, College of Bioresources and Agriculture National Taiwan University Taipei 10617 Taiwan, ROC; ^5^ National Taiwan University Veterinary Hospital, College of Bioresources and Agriculture National Taiwan University Taipei 10672 Taiwan, ROC; ^6^ Lifecare Animal Hospital Taipei 11271 Taiwan, ROC; ^7^ Uni‐Pharma Co‐Ltd. Taipei 11494 Taiwan, ROC; ^8^ Thoughtful Animal Hospital Taoyuan 33820 Taiwan, ROC; ^9^ Department of Veterinary Medical Sciences The University of Tokyo Tokyo 113‐8657 Japan

**Keywords:** biomarker, DR‐70, fibrinogen‐fibrin degradation products, tumor detection

## Abstract

**Background:**

Tumor biomarkers have used widely in clinical oncology in human medicine. Only a few studies have evaluated the clinical utility of tumor biomarkers for veterinary medicine. A test for fibrinogen and fibrin degradation products (DR‐70) has been proposed as an ideal biomarker for tumors in humans. The clinical value of DR‐70 for veterinary medicine however has yet to be determined.

**Objectives:**

Investigate the diagnostic value of DR‐70 concentrations by comparing them between healthy dogs and dogs with tumors.

**Animals:**

Two hundred sixty‐three dogs with different types of tumors were included. Sixty healthy dogs also were recruited for comparison.

**Methods:**

The DR‐70 concentrations were measured in all recruited individuals by ELISA. Clinical conditions were categorized based on histopathology, cytology, ultrasound examination, radiology, clinical findings, and a combination of these tests.

**Results:**

The median concentration of DR‐70 was 2.130 ± 0.868 μg/mL in dogs with tumors, which was significantly higher than in healthy dogs (1.202 ± 0.610 μg/mL; *P* < .0001). With a cut‐off of 1.514 μg/mL, the sensitivity and specificity of DR‐70 were 84.03% and 78.33%, respectively. The area under curve was 0.883. The DR‐70 concentration can be an effective tumor biomarker in veterinary medicine.

**Conclusions and Clinical Importance:**

Increased DR‐70 concentrations were not affected by tumor type, sex, age, or body weight. However, in dogs with metastatic mast cell tumors and oral malignant melanoma, DR‐70 concentrations were significantly increased. Additional studies, including more dogs with nonneoplastic diseases, are needed to further evaluate the usefulness of DR‐70 as a tumor biomarker.

**Abbreviations**
AUC: area under the ROC curve
BCL: B‐cell lymphoma
EDTA: ethylenediaminetetraacetic acid
ELISA: enzyme‐linked immunoassay
FDPs: fibrinogen‐fibrin degradation products
HRP: horseradish peroxidase
MCTs: mast cell tumors
MGT: mammary gland tumor
NSAIDs: non‐steroidal anti‐inflammatory drugs
ROC: receiver‐operator characteristics
TCC: transitional cell carcinoma
TCL: T‐cell lymphoma
TMB: tetramethylbenzidine
WHO: World Health Organization

## 1 INTRODUCTION

Neoplastic diseases affect a substantial proportion of dogs, with >50% of dogs >10 years of age developing tumors.^1^ However, tumors in dogs often are not identified until more advanced stages,^2^, ^3^ complicating medical intervention for affected animals.^4^, ^5^ Early detection of tumors is crucial, and biomarkers have emerged as valuable indicators for tumor detection and monitoring of tumor progression.^6^, ^7^ Although early tumor diagnosis using biomarkers has gained attention in veterinary medicine,^8^ the translation of biomarkers into successful clinical use has been limited.^9^ In previous studies in humans, fibrinogen‐fibrin degradation products (FDPs), specifically DR‐70, have been strongly associated with gastric and colorectal tumorigenesis, making them a potential method for early tumor detection.^10^, ^11^

The clinical relevance of hemostatic abnormalities in tumorigenesis and the malignant process remains controversial. Coagulation and fibrinolysis activation within tumors have been proposed as potential mechanisms that promote tumor growth and metastasis.^12^ Another possible mechanism involves tumor cells releasing plasmin to facilitate invasion and metastasis by promoting fibrinolysis. The cleavage of fibrinogen leads to the release of DR‐70 fragments.^11^ These processes suggest that tumors are a source of DR‐70, and higher DR‐70 concentrations positively correlate with tumor development.

Fibrinogen degradation products and fibrin degradation products (1 of which is D‐dimer) must be considered differently. Fibrinogen degradation products (fragments X, D, Y, and E) are produced from fibrinogen, whereas fibrinolysis creates more complex fragments called X‐oligomers, which contain the D and E fragments in various sequences.^13^, ^14^ Only fibrin polymers, mediated by factor XIII cross‐linking, will produce fragments containing covalent bonds between 2 D domains (D‐dimer). Therefore, D‐dimer is a specific fibrin degradation product that results from fibrinolysis. Affected patients generate fibrinogen and fibrin degradation products to restrict cancer cells, and the DR‐70 assay measures both fibrinogen and fibrin degradation.^10^, ^15^, ^16^ The commercial D‐dimer immunoassay specifically determines the epitope on the degradation products of factor XIII‐cross‐linked fibrin, but fails to detect fibrinogen degradation products.^13^, ^14^ As an ideal tumor biomarker, tests that only measure D‐dimer will miss up to half of the actual FDPs produced by tumors.^17^ Therefore, determination of DR‐70 concentrations has gained more attention in human medical oncology.

The DR‐70 concentrations have been extensively studied in human clinical medicine as a diagnostic tool for tumor detection in various malignancies, including lung,^18^ tongue,^19^ gastrointestinal tract,^20^ colorectum,^11^ esophagus,^21^ and ovary.^22^ These studies indicate significant correlation between increased DR‐70 concentrations and tumor formation across multiple types of tumors. However, the accuracy and utility of DR‐70 as a tumor biomarker in veterinary medicine remain unknown. Additionally, no previous publication in veterinary oncology has explored DR‐70 concentrations across different tumor types stratified by sex, age, and breed. Therefore, we aimed to investigate the diagnostic potential of plasma DR‐70 concentrations in dogs with tumors compared to healthy individuals. Furthermore, we sought to determine DR‐70 concentrations among different tumor types and assess the categorization of DR‐70 concentrations based on other variables in tumor‐bearing dogs.

## 2 MATERIALS AND METHODS

### 2.1 Sample collection and processing

Our study was approved by the Institutional Animal Care and Use Committee of National Taiwan University, Taipei, Taiwan (protocol code: IACUC No. NTU110‐EL‐00096). All samples were collected with informed client consent, and all experiments followed relevant guidelines. For the healthy controls (defined as controls), canine plasma samples from active patients without neoplastic disease or healthy dogs at the National Taiwan University Veterinary Hospital and Veterinary Medical Center, the University of Tokyo, were collected with owner consent. Dogs were designated as controls based on results from a client questionnaire and physical examination by attending veterinarians. For tumor‐bearing dogs (defined as patients), individuals of any age, sex, or breed were enrolled in this study. However, patients with concurrent diseases unrelated to neoplasia and deemed to be influencing the diagnosis were not eligible. Tumor types were diagnosed by histopathology, cytology or both for all patients.

For all participants, regular physical examinations, evaluation of superficial lymph nodes, and blood evaluation (CBC and serum biochemistry) were evaluated. No dogs received non‐steroidal anti‐inflammatory drugs (NSAIDs) or corticosteroids for 14 days before blood collection. Participants previously receiving chemotherapy (patients), anti‐coagulant drugs, or blood transfusion also were excluded. All dogs were fasted for at least 8 hours before blood collection, and a minimum of 0.5 mL of plasma was collected from each dog. Samples were drawn from a cephalic or jugular vein into an EDTA tube (BD Vacutainer, Franklin Lakes, New Jersey) and immediately centrifugated at 450 *g* for 10 minutes at 4°C. Next, supernatants were collected and centrifugated at 16 000 *g* for 10 minutes at 4°C. After centrifugation plasma samples were collected, transferred into new tubes, and frozen at −80°C until analysis.

### 2.2 Patient selection and evaluation

We collected clinical data, including breed, age, reproductive status, and tumor diagnosis. The recruited dogs were further allocated into different types of tumors according to the histopathological or cytological analysis. Of these patient dogs, cases with ≥2 types of tumors and medical records with limited information were categorized as the “other” group. For dogs with lymphoma, the immunophenotype was determined by flow cytometry or immunohistochemistry.^23^ Mast cell tumors (MCTs) were categorized by recorded grade according to the clinical staging system proposed by the World Health Organization (WHO).^24^ Dogs with regional or systemic metastasis were classified as stage II or above.

### 2.3 Measurement of FDP using DR‐70 ELISA kit

According to the manufacturer's instructions, plasma DR‐70 concentrations were measured using a commercial ELISA kit (DR‐70 ELISA, Uni‐pharma Inc., Taipei, Taiwan).^10^, ^16^ The retail kit contained a microwell plate of 96‐well strips coated with affinity‐purified rabbit anti‐DR‐70 polyclonal antibodies, a horseradish peroxidase (HRP)‐labeled detection antibody, tetramethyl benzidine (TMB) substrates, diluent solution, stop solution, wash buffer, low limits of quantification control, high limits of quantification control, and 5 calibrators. Briefly, the plasma samples were diluted 250‐fold using the diluent solution. Next, the diluted samples were added to the antibody‐coated wells in a volume of 100 μL in duplicate. After an incubation period of 30 minutes at room temperature, the microwells were washed 5 times with 1× wash buffer. The washed microwells then were tapped on clean absorbent paper. Next, the HRP‐conjugated antibodies were added into each well in a volume of 100 μL and incubated for another 30 minutes at room temperature. After incubation, the microwells were washed 5 times with 1× wash buffer. Next, TMB substrate solution was added to each well and incubated in the dark for 15 minutes. Lastly, the reactions were stopped by adding 100 μL stop solution to each well. The end‐point of the test was read by measuring the absorbance at 450 nm using a microplate reader (SpectraMax M5 Microplate Reader, San Jose, CA, USA). A standard curve was generated from the absorbances of the 5 calibrators provided in the commercial kit. The DR‐70 concentrations of plasma were quantified by interpolation from the standard curve.

### 2.4 Preparation and determination of canine fibrinogen degradation products

Canine fibrinogen degradation products (cFDPs) were prepared according to a previous study.^25^ Briefly, 150‐200 mg of canine fibrinogen (Abcam, catalog no.: ab93006, Taipei, Taiwan) was dissolved in 10 mL of 20 mmol/L triethanolamine buffer. Degradation of fibrinogen was induced by incubating the mixture in a water bath at 37°C for 2 hours, with the addition of 10 casein units of plasmin per 100 mg of fibrinogen. The fibrinogenolysis process was halted by adding 2000 kilo international units (KIU) of aprotinin per milliliter. The concentration of cFDPs was quantified by bicinchoninic acid (BCA) protein assay (Bio‐Rad Laboratories, Hercules, California). As shown in Figure S1, a standard curve was generated from the absorbances of the cFDPs (red line), which shared high similarity with the 5 calibrators provided in the commercial kit (blue line). These results indicate that the commercial kit also can specifically recognize canine cFDPs.

### 2.5 Statistical analysis

GraphPad Prism Software 9.0 (GraphPad Software, INC, La Jolla, California, USA) was used for all statistical analyses. Data were tested for normal distribution using the D'Agostino & Pearson test. Because results were not normally distributed, nonparametric tests were conducted for data comparisons. The Mann‐Whitney *U* test was used for data sets containing only 2 populations, such as controls compared with patients. The Kruskal‐Wallis test for repeated measures with Dunn's multiple comparisons test was used for data sets where various groups were analyzed. To evaluate the diagnostic performance of the DR‐70 assay, receiver‐operator characteristics (ROC) curves were generated, along with the area under the ROC curve (AUC). The sensitivities and specificities of the DR‐70 results were calculated at various threshold concentrations. Youden's index (*J*), a measurement for evaluating biomarker effectiveness,^26^ was used to determine the cut‐off for calculating sensitivity and specificity. The optimal cut‐off is defined as the maximum value of *J*.^27^, ^28^ Data are presented as the median and interquartile range (IQR, range from the 25th to the 75th percentile). Statistical difference was defined as a *P*‐value <.05.

## 3 RESULTS

### 3.1 Clinical characteristics

We enrolled 60 healthy control dogs with a median age of 4 years (range, 1.3‐11 years). Mountain cur dogs were the most common breed encountered (n = 12), followed by 9 mixed breed dogs, 6 beagles, 4 border collies, 4 Maltese dogs, 4 miniature dachshunds, 3 Irish setters, 3 miniature poodles, 3 Pembroke Welsh corgi dogs, 3 miniature schnauzers, 2 French bulldogs, 2 Shiba inu dogs, 2 shih tzu dogs and 1 each of chihuahua, golden retriever, and standard poodle. Reproductive status consisted of 16.7% intact males (n = 10), 33.3% castrated males (n = 20), 21.7% intact females (n = 13), and 28.3% spayed females (n = 17). Detailed signalments and DR‐70 concentrations of the healthy control dogs are summarized in Table S1.

Tumor types of individual dogs were diagnosed by histopathology or cytology. Of the 263 patients in the study, there were 116 lymphoma cases, 17 MCT cases, 12 melanoma cases, and several additional tumor types. Among patients, age was unknown for 46 dogs, with 217 ranging from 1 to 17 years of age (median, 9 years; mean, 8.9 years). The diagnosis of tumor types and detailed characteristics of all patients are summarized in Table S2.

### 3.2 Significantly increased DR‐70 concentrations in tumor‐bearing dogs with high diagnostic power

**FIGURE 1:**
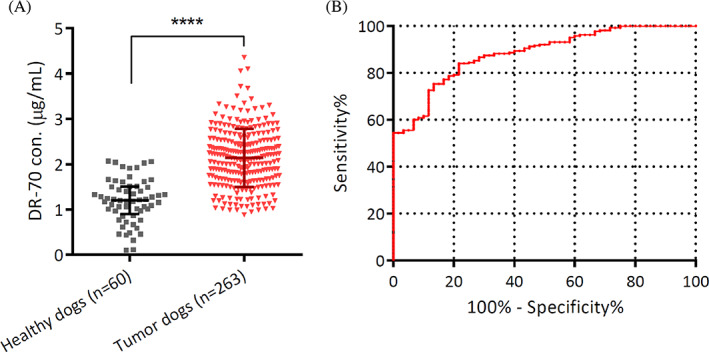
Increased DR‐70 concentrations in the plasma of dogs with tumors. (A) The median concentrations of DR‐70 in healthy controls and dogs with neoplasms were 1.202 ± 0.610 and 2.130 ± 0.868 μg/mL, respectively. (B) ROC curve demonstrates the area under the ROC curve (AUC) for all dogs with tumors compared to healthy animals. The AUC is 0.883 under a 95% confidence interval of 0.842‐0.923, with a significant difference (*P* < .0001). Data were described as median ± interquartile range and analyzed by the Mann‐Whitney *U* test. *P* values <.05 were considered significant. ****P < .0001.

The DR‐70 concentrations were measured in duplicate for all recruited dogs. As shown in Figure 1A, the median DR‐70 concentration in patients (2.130 ± .868 μg/mL) was significantly higher than in healthy controls (1.202 ± 0.610 μg/mL; *P* < .0001). The ROC assessed the ability of DR‐70 to distinguish clinical cases from healthy controls, and the AUC was evaluated. The AUC was 0.883 with a 95% confidence interval (CI) of 0.833 to 0.923, which indicated ideal diagnostic performance of DR‐70 to discriminate between dogs with tumors and healthy controls (Figure 1B; *P* < .0001).

To elucidate the impact of age (>7 years), sex, and reproductive status of these healthy dogs, we divided the healthy dogs into different groups according to the variables. The results showed that there were no significant differences between dogs stratified by age (Figure S2A; <7 years, n = 46, 1.177 ± 0.495 μg/mL; ≥7 years, n = 14, 1.472 ± 0.857 μg/mL; *P* > .05). Dogs were divided into 2 groups based on sex (male, n = 30, 1.217 ± 0.582 μg/mL; female, n = 30, 1.190 ± 0.607 μg/mL). The concentrations of DR‐70 in males and females were not significantly different (Figure S2B; *P* > .05). Next, patients were separated into 2 groups based on reproductive status (intact, n = 23, 1.170 ± 0.339 μg/mL; castrated or spayed, n = 27, 1.225 ± 0.697 μg/mL). As shown in Figure S2C, no significant difference was found based on reproductive status (*P* > .05).

### 3.3 Increased DR‐70 concentrations in dogs with metastatic MCTs and oral malignant melanomas

**FIGURE 2:**
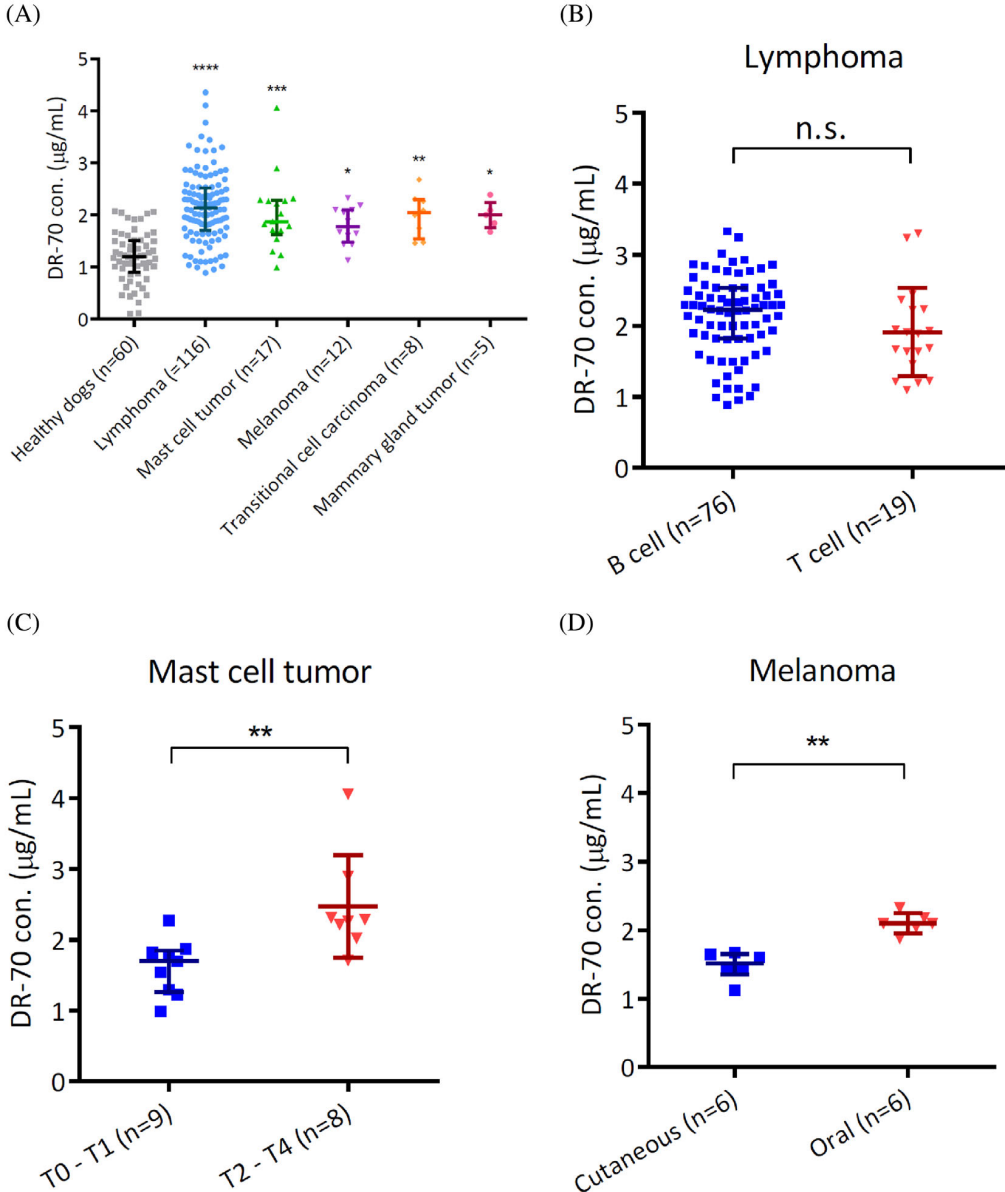
DR‐70 concentrations across multiple tumor types relative to the healthy cases. (A) DR‐70 concentrations were categorized by various common tumor types, such as lymphoma (n = 116), mast cell tumor (n = 17), melanoma (n = 12), TCC (n = 8), and mammary gland tumor (n = 5). (B) DR‐70 concentrations for lymphoma cases by immunophenotyping (B‐cell or T‐cell lymphoma). (C) DR‐70 concentrations for dogs with mast cell tumors according to grades (T0‐T1 or T2‐T4). (D) DR‐70 concentrations for melanoma cases by location (cutaneous or oral region). Data were described as median ± interquartile range and analyzed by the Mann‐Whitney *U* or Kruskal‐Wallis test. *P* values <.05 were considered to indicate statistical significance. *P < .05; **P < .01; ***P < .001; ****P < .0001; n.s., no significant difference.

When all cases with tumors were considered together compared to healthy controls, the DR‐70 concentrations significantly increased in the patient group. Plasma DR‐70 concentrations were evaluated for tumors with at least 5 cases each. We then analyzed the DR‐70 concentrations of dogs with the most common types of tumors, including lymphoma (n = 116), MCT (n = 17), melanoma (n = 12), transitional cell carcinoma (TCC, n = 8), and mammary gland tumor (MGT, n = 5). The DR‐70 concentrations of lymphoma, MCT, melanoma, TCC, and MGT were 2.135 ± 0.820, 1.873 ± 0.660, 1.777 ± 0.620, 2.038 ± 0.756, and 1.999 ± 0.481 μg/mL, respectively (Figure 2A). These concentrations were significantly higher than those of the healthy controls, whereas differences among tumor types were not significant (*P* > .05).

We next evaluated whether increased DR‐70 concentrations were present for B‐cell (BCL) and T‐cell (TCL) lymphoma. Immunophenotyping information was available for 95 cases, and DR‐70 concentrations were compared between 2 immunophenotype groups and healthy control dogs. The DR‐70 concentrations were significantly increased in dogs with BCL and TCL compared to healthy controls. The median DR‐70 concentrations for BCL and TCL were 2.223 ± 0.713 μg/mL (n = 76) and 1.891 ± 0.770 μg/mL (n = 19), respectively. However, no significant difference was found in these 2 populations (*P* > .05; Figure 2B). The MCTs were divided by recorded grade, and this information was available for 17 dogs with MCTs. Dogs with grade 0 and 1 MCTs (n = 9, 1.689 ± 0.586 μg/mL) had lower DR‐70 concentrations compared to those with grades 2, 3, and 4 (n = 8, 2.275 ± 0.680 μg/mL; *P* < .01; Figure 2C). Among melanoma cases, 2 main groups were recorded: cutaneous (n = 6) and oral (n = 6) malignant melanomas. The cutaneous melanoma group had 4 benign cases; the other 2 were not characterized. All of the oral melanoma cases were diagnosed as malignant tumors. The DR‐70 concentrations were significantly lower in dogs with cutaneous melanoma (n = 6, 1.518 ± 0.297 μg/mL) compared to those with oral melanoma (n = 6, 2.096 ± 0.215 μg/mL; *P* < .01; Figure 2D). Collectively, dogs with metastatic MCTs or oral malignant melanoma had increased DR‐70 concentrations. These results indicate that DR‐70 concentrations are positively correlated with tumor malignancy.

### 3.4 Plasma DR‐70 concentrations in dogs with tumors are not affected by sex, age, or body weight

**FIGURE 3:**
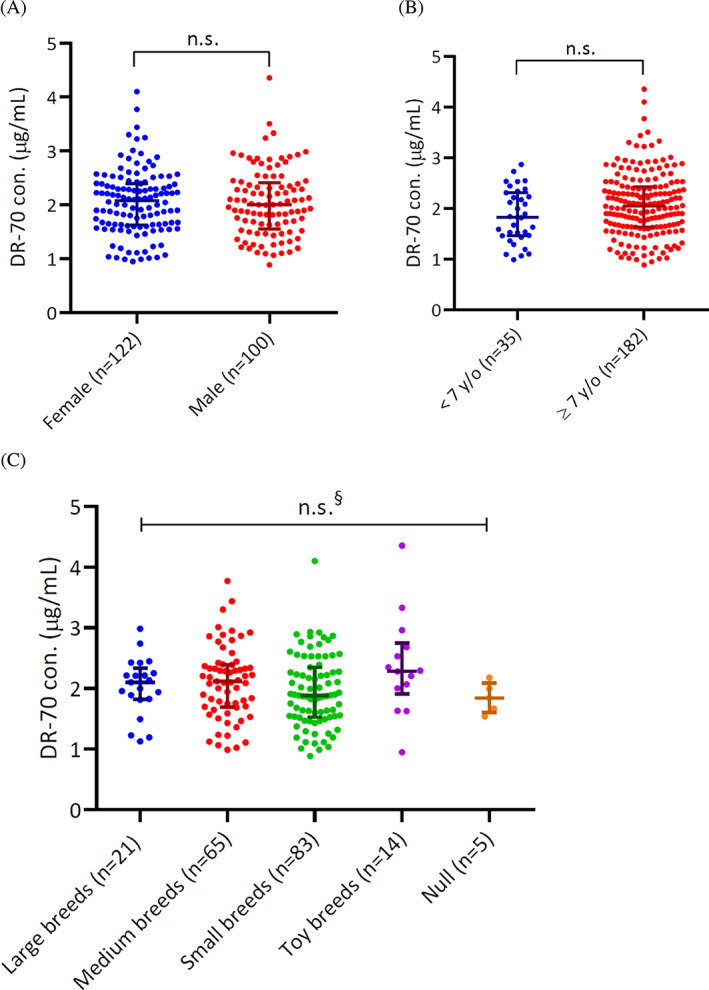
Comparisons of DR‐70 concentrations in dogs with tumors by sex, age, and breeds. DR‐70 concentrations for dogs with tumors were not significantly different according to (A) sex (females or males), (B) age (< or >7 years old), and (C) breeds (large, medium, small, or toy breeds). Data were described as median ± interquartile range and analyzed by the Mann‐Whitney *U* or Kruskal‐Wallis test. *P* values <.05 were considered significant. n.s., no significant; §, no significant difference among the 5 groups.

Among the patient group, 222 dogs had a reported sex, and 217 had a reported age. A total of 183 dogs had a registered breed. Detailed DR‐70 concentrations, including minimum, maximum, mean, and median, for dogs categorized by sex, age, and species are shown in Table 1. First, dogs were divided into 2 groups based on sex (female, n = 122, 2.076 ± 0.767 μg/mL; male, n = 100, 2.002 ± 0.886 μg/mL). The concentrations of DR‐70 in males and females were not significantly different (Figure 3A; *P* > .05). Next, patients were separated into 2 groups based on age (<7 years, n = 35, 1.825 ± 0.854 μg/mL; ≥7 years, n = 182, 2.049 ± 0.792 μg/mL). Both patient groups had significantly higher median DR‐70 concentrations than healthy controls. In contrast, no significant difference in DR‐70 concentrations was found between these 2 populations (Figure 3B; *P* > .05). Lastly, dogs with tumors were allocated into 5 groups based on body weight as defined by The American Kennel Club (large breed, n = 21, 2.103 ± 0.514 μg/mL; medium breed, n = 65, 2.118 ± 0.700 μg/mL; small breed, n = 83, 1.886 ± 0.820 μg/mL; toy breed, n = 14, 2.287 ± 0.841 μg/mL). Detailed DR‐70 concentrations for each breed are summarized in Table 2. When each group was evaluated, no significant differences were found among these groups of tumors in any category (Figure 3C; *P* > .05 for all comparisons). These results indicate that sex, age, and size did not affect DR‐70 concentrations in tumor‐bearing dogs.

**TABLE 1:** Comparisons of DR‐70 concentrations by sex, age, and breed in dogs with tumors.

	Number	Minimum	Maximum	Mean	Median	*P* value
Sex						.83
Male	100	0.8840	4.355	2.042	2.002	
Female	122	0.9480	4.103	2.049	2.076	
Age (years)						.05
<7‐year‐old	35	0.9890	2.868	1.852	1.825	
>7‐year‐old	182	0.8840	4.355	2.080	2.049	
Breed						>.05
Large breeds	21	1.129	2.987	2.030	2.103	
Medium breeds	65	0.9881	3.773	2.102	2.118	
Small breeds	83	0.8840	4.103	1.950	1.886	
Toy breeds	14	0.9480	4.355	2.376	2.287	
Null	5	1.538	2.179	1.846	1.845	

**TABLE 2:** DR‐70 concentrations are categorized by breeds of dogs with tumors.

	DR‐70 concentrations	Case number (percentage)	Breed weight chart (kg)^a^
Large breeds^a^	2.103 (0.423)	21 (11.2%)	24.95‐38.56
Old English Sheepdogs	1.954	1 (4.8%)	27.22‐45.36
Retrievers (Golden)	2.172 (0.476)	18 (85.7%)	24.95‐34.02
Retrievers (Labrador)	1.505 (0.311)	2 (9.5%)	24.95‐36.92
Medium breeds	2.118 (0.692)	65 (34.6%)	15.88‐29.48
Border Collies	1.530 (0.778)	5 (7.7%)	13.61‐24.95
Bulldogs	1.486 (0.022)	2 (3.1%)	18.14‐22.68
Mixed (Taiwan dog)	2.206 (0.639)	53 (81.5%)	11.79‐18.14
Siberian Huskies	2.100 (0.465)	5 (7.7%)	15.88‐27.22
Small breeds	1.886 (0.782)	83 (44.1%)	3.18‐15.88
Beagle	1.757 (0.405)	10 (2.0%)	9.07‐13.61
French Bulldog	1.760 (0.573)	4 (4.8%)	<12.70
Fox Terriers	2.086	1 (1.2%)	2.72‐8.16
Miniature Dachshunds	1.720 (1.057)	10 (12.0%)	<4.99
Miniature Poodle	1.951 (0.705)	20 (24.1%)	4.54‐6.80
Miniature Schnauzers	1.929 (0.709)	21 (25.3%)	4.99‐9.07
Pembroke Welsh Corgi	2.633 (0.767)	6 (7.2%)	<12.7‐13.61
Pug	2.690	1 (1.2%)	6.35‐8.16
Shiba Inu	1.395 (0.486)	6 (7.2%)	7.71‐10.23
Shih Tzu	2.143 (0247)	2 (2.4%)	4.08‐7.26
West Highland White Terriers	1.637	1 (1.2%)	6.80‐9.07
Whippet	1.876	1 (1.2%)	11.34‐18.14
Toy breeds	2.287 (0.625)	14 (7.4%)	0.91‐4.08
Chihuahua	1.627 (0.528)	3 (21.4%)	<2.72
Maltese	2.440 (0.663)	10 (71.4%)	<3.18
Yorkshire Terrier	2.292	1 (1.1%)	3.18
Null	1.845 (0.332)	5 (2.7%)	

*Note*: Data were presented as median (interquartile range).

^a^ American Kennel Club (AKC) defined the breed weight chart and breeds.

### 3.5 High sensitivity and specificity of DR‐70 for tumor diagnosis at an optimal concentration of 1.514 μg/mL

Additional diagnostic test variables were calculated for DR‐70. The diagnostic sensitivity and specificity of the DR‐70 varied by the selected cut‐off. According to the results, with cut‐offs extending from 1.494 to 1.536 μg/mL, sensitivity was between 82.13% and 84.79%, whereas specificity was between 73.33% and 78.33% (Table 3). Based on the maximum value of *J*, an optimal cut‐off of 1.514 μg/mL with a sensitivity of 84.03% (95% CI, 79.03%‐88.24%) and specificity of 78.33% (95% CI, 65.80%‐87.93%) was selected. These results indicate that at a concentration of 1.514 μg/mL, DR‐70 has a high potential to be designated as a biomarker for distinguishing dogs with and without tumors.

**TABLE 3:** Sensitivity and specificity of DR‐70 for diagnosis of tumors at different cut‐off concentrations.

DR‐70 cut‐off (μg/mL)	Sensitivity (%)	95% CI (%)	Specificity (%)	95% CI (%)
>1.494	84.79	79.87‐88.91	73.33	60.34‐83.93
>1.497	84.41	79.45‐88.57	73.33	60.34‐83.93
>1.503	84.41	79.45‐88.57	75.00	62.14‐85.28
>1.508	84.03	79.03‐88.24	75.00	62.14‐85.28
>1.511	84.03	79.03‐88.24	76.67	63.96‐86.62
>1.514	84.03	79.03‐88.24	78.33	65.80‐87.93
>1.518	83.65	78.62‐87.91	78.33	65.80‐87.93
>1.523	83.27	78.20‐87.57	78.33	65.80‐87.93
>1.528	82.89	77.78‐87.24	78.33	65.80‐87.93
>1.532	82.51	77.37‐86.90	78.33	65.80‐87.93
>1.536	82.13	76.95‐86.56	78.33	65.80‐87.93

Abbreviation: CI, confidence interval.

### 3.6 Increased DR‐70 concentrations in dogs with inflammation and acute diseases

**FIGURE 4:**
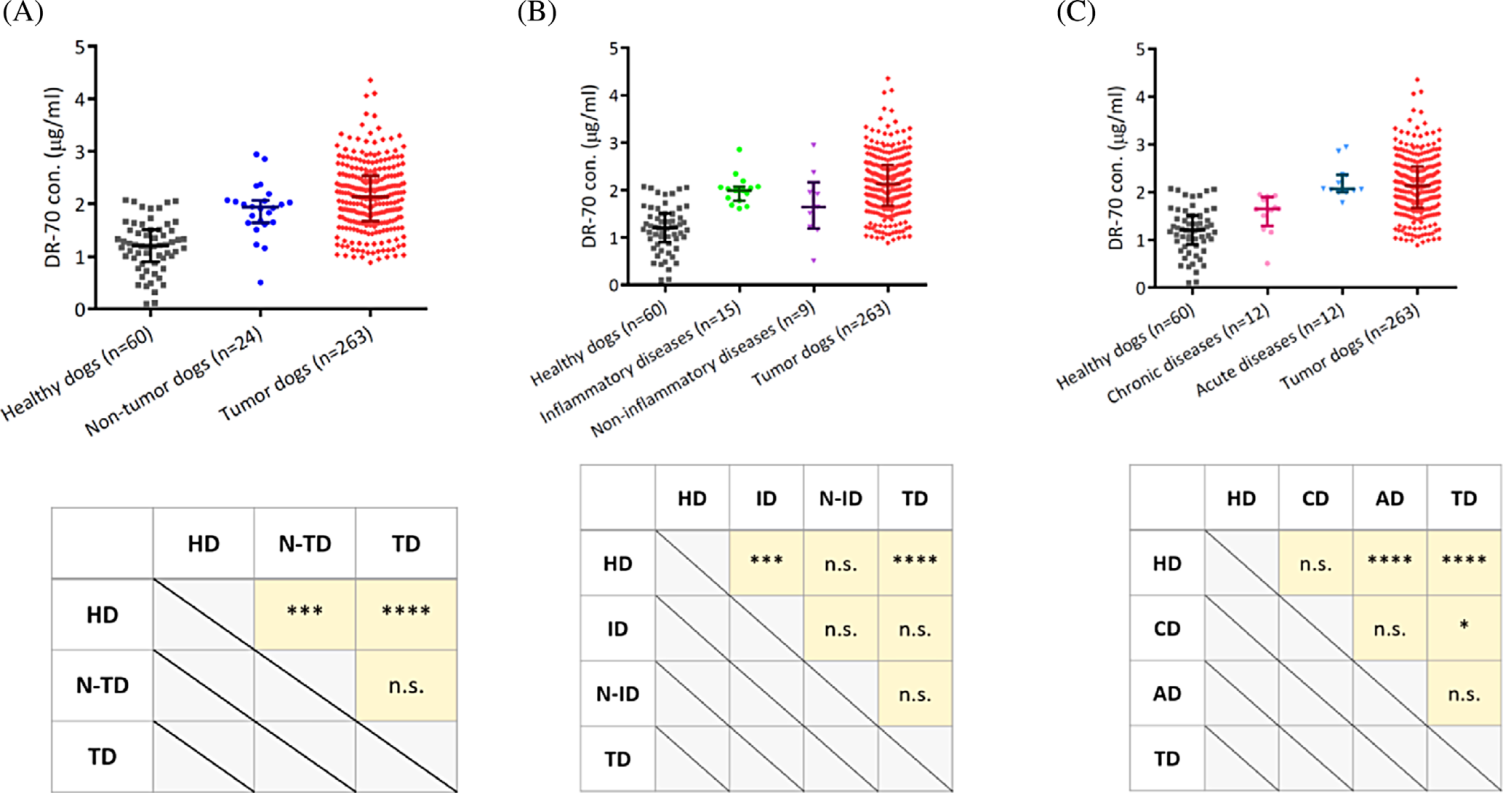
Comparisons of DR‐70 concentrations in dogs with tumors and with nonneoplastic diseases. (A) Statistical analysis of DR‐70 concentrations stratified by nonneoplastic diseases, (B) inflammatory status, (C) and disease progression compared with healthy and tumor dogs. Data were described as median ± interquartile range and analyzed by the Kruskal‐Wallis test. *P* values <.05 were considered significant. n.s., no significant difference; *P < .05; ***P < .001; ****P < .0001. AD, acute diseases; CD, chronic diseases; HD, healthy dogs; ID, inflammatory diseases; N‐ID, non‐inflammatory diseases; N‐TD, non‐tumor dogs; TD, tumor dogs.

To elucidate DR‐70 concentrations in dogs without neoplasia, we enrolled 24 patients without tumors at National Taiwan University Veterinary Hospital (Table S3). The DR‐70 concentrations were significantly increased in dogs with tumors (*P* < .0001) and in non‐tumor‐bearing dogs (n = 24, 1.937 ± 0.424 μg/mL; *P* < .001) compared to controls. No difference was found between dogs with tumors and non‐tumor‐bearing dogs (Figure 4A). We further allocated the non‐tumor‐bearing dogs into 2 groups (inflammatory or non‐inflammatory diseases) according to general evaluation by clinical veterinarians (Figure 4B). The DR‐70 concentrations in dogs with inflammation (n = 15, 1.987 ± 0.295 μg/mL; *P* < .001) were significantly higher than those in the controls, but were not different compared to dogs with tumors. In dogs without inflammation (n = 9, 1.639 ± 0.970 μg/mL), DR‐70 concentrations were not different from concentrations in controls, dogs with inflammation, and dogs with tumors. As shown in Figure 4C, the non‐tumor‐bearing dogs were divided into 2 populations again, and stratified by disease phase (acute or chronic). Increased DR‐70 concentrations were found in dogs with acute diseases versus controls (n = 12, 2.064 ± 0.366 μg/mL; *P* < .0001), and not significantly different from dogs with tumors. Dogs with chronic diseases exhibited relatively low DR‐70 concentrations (n = 12, 1.646 ± 0.597 μg/mL) compared to those with tumors (*P* < .05). Notably, no significant difference was found between dogs with acute and chronic diseases (*P* = .05). Increased DR‐70 concentrations found in dogs with inflammatory diseases might result from an acute phase response. We speculated that DR‐70 concentrations might return to normal after resolution of inflammation.

## 4 DISCUSSION

Developing biomarkers to diagnose tumors is essential in human and veterinary medicine. We demonstrated that DR‐70 concentrations are practical biomarkers for distinguishing dogs with or without tumors. In our study, DR‐70 concentrations were significantly higher in the tumor‐bearing dogs than in the healthy controls. Plasma DR‐70 concentrations were further analyzed for certain types of tumors (n ≥ 5 for each type of tumor), but no significant differences were found among these groups. Notably, dogs with metastatic MCT or oral malignant melanoma had higher concentrations of DR‐70 than did those with local MCT or cutaneous melanoma. These findings indicate that DR‐70 concentration is a useful diagnostic biomarker in dogs with tumors.

With regard to the similarity between tumors in humans and dogs, as canine genome sequencing became available, humans and dogs were found to be more genetically similar than humans and rodents.^29^ Regarding cancer‐related genes, several studies have shown high similarity between humans and dogs, such as mTOR, RBRCA, retinoblastoma, p16, p53, and other genes.^30^, ^31^ In addition, tumors in humans and dogs have a high degree of histopathological similarity, and the pattern of distant metastasis also is similar in some tumor types. For example, melanoma is prone to lung metastasis in dogs.^32^ In addition, many similarities exist between human breast cancer and mammary cancer in dogs. For example, human and canine triple‐negative breast tumors have more rapid recurrence times and higher recurrence rates than do endocrine receptor‐positive breast tumors. There is also a higher rate of invasion of surrounding tissues and metastases. These characteristics also are found in MGTs in dogs.^33^ This similarity likely is because the interaction between tumor growth and the immune system is identical, and even relapse after treatment and drug resistance are similar in dogs.^34^ In addition, humans and dogs inhabit the same environment for long periods, making environmental carcinogens a possible commonality for tumors in both humans and dogs.^35^ In the past, experimental data from dogs could guide the treatment of cancers in humans and cancer research. As cancer treatment in humans has made substantial advancements, these advances can be used to inform cancer treatment in dogs, with tumor markers used for humans also being utilized for cancer diagnosis in dogs.

Few studies have described increased DR‐70 concentrations in dogs with tumors. Several studies focused on the upstream product, fibrinogen, are widely reported in veterinary medicine. One study reported abnormal coagulation parameters in dogs with tumors, such as hyperfibrinogenemia.^36^ Dogs with tumors often present with abnormal hemostatic parameters, including increased fibrinogen concentrations.^37^ However, these previous studies focused on reporting abnormal coagulation tests or hemostatic alterations in dogs with neoplastic diseases, and the clinical relevance and utility of DR‐70 concentrations were rarely described. To the best of our knowledge, our study is the first to report on the clinical relevance of DR‐70 in veterinary oncology.

Our study found increased DR‐70 concentrations in dogs with tumors and analyzed these in certain tumors. The DR‐70 concentrations of dogs with BCL and TCL were not significantly different. The underlying mechanisms of this phenomenon remain unknown. One possible explanation is the inequality of sample numbers between BCL (n = 76) and TCL (n = 19). Verifying these findings with additional cases of TCL would be useful. Standard immunophenotyping (CD3/CD5 positivity for TCL or CD19/CD21 positivity for BCL) failed to distinguish less aggressive lymphoma from more malignant tumors. For example, in dogs with TCL, some samples were low‐grade (indolent), advanced TCL, or different subtypes of malignancies. However, because of the restrictions of our study's retrospective design, we could not analyze the DR‐70 concentrations among different subtypes of TCL. Previous veterinary studies have shown that hemostatic parameters are higher in aggressive tumors.^38^, ^39^, ^40^, ^41^ Hence, we speculate that DR‐70 concentrations might increase more in malignant subtypes of lymphoma than in other lymphoma phenotypes. If aggressiveness was to be characterized, it is expected that DR‐70 concentrations would be affected in specific populations. Lastly, although it is also possible that DR‐70 concentrations in dogs with BCL and TCL are not significantly different, additional studies are needed to expand on these considerations.

Our study achieved a sensitivity of 84.03% and a specificity of 78.33% in differentiating tumor‐bearing dogs from healthy controls using DR‐70 concentration at a cut‐off of 1.514 μg/mL. Among 195 patients, 38 cases were below this cut‐off, including 3 of 9 dogs with T0‐T1 MCT and 3 of 6 dogs with cutaneous melanoma. Notably, all 8 dogs with T2‐T4 MCT and 6 cases of oral malignant melanoma had DR‐70 concentrations >1.514 μg/mL. This finding was expected because DR‐70 concentrations correlate with tumor grading and malignancy. Furthermore, dogs with metastatic MCTs (T2‐T4) had significantly higher plasma concentrations of DR‐70 than those without metastasis (T0‐T1). We used the WHO grading system to grade these samples. Thus, additional investigations are recommended to verify that similar differences are found when another grading system, such as a 2‐tier histologic grading system, is applied.

Furthermore, we found that DR‐70 concentrations might positively correlate with overall survival time in dogs with MCTs. Dogs with T0‐T1 MCTs exhibited longer median overall survival time (n = 5; median, 569 days; range, 126‐1065 days) than did those with T2‐T4 MCTs (n = 5; median, 222 days; range, 108‐506 days). However, we did not present data this because of missing medical records and different medical interventions in these dogs. Therefore, well‐designed studies with larger sample sizes are needed to clarify this finding. We also found that dogs with oral malignant melanoma had increased DR‐70 concentrations, significantly higher than those with cutaneous melanoma. Almost all cases of cutaneous melanomas were benign, whereas melanomas in the oral cavity were malignant in our study. This finding indicates the possibility of using DR‐70 concentrations to discriminate between less aggressive melanoma and more malignant melanoma. However, the medical records did not report the malignant status of 2 cases with cutaneous melanomas. Therefore, additional studies with detailed histopathological examinations for each melanoma case should be performed to support our findings.

Our study had some limitations. First, this preliminary investigation involved several types of tumors in dogs, and additional studies that focus on certain types of tumors are needed. Furthermore, the retrospective design of our study restricted our findings. Cohort and case‐control prospective studies would be useful. Although more investigations are needed to address these issues, our study did illustrate that DR‐70 has excellent potential to be considered as a tumor biomarker in veterinary medicine.

In conclusion, DR‐70 concentrations were increased in the investigated patients, and dogs with metastatic MCTs or oral malignant melanoma had higher concentrations of DR‐70. These results indicate that DR‐70 could be a useful tumor biomarker. Additional investigations are recommended for confirmation of the accuracy and clinical relevance of DR‐70 as a tumor biomarker.

## CONFLICT OF INTEREST DECLARATION

Chun‐Hung Wu and Chin‐Hao Hu are compensated as consultants to Uni‐Pharma Co‐Ltd. Uni‐Pharma Co‐Ltd employs Chueh‐Ling Lu and Chiao‐Lei Cheng. Yu‐Shan Wang is the general manager at Uni‐Pharma Co‐Ltd and holds vested equity in Uni‐Pharma Co‐Ltd. Yu‐Shan Wang is also an inventor of multiple patent applications covering technologies for cancer diagnostics. No other authors declare a conflict of interest.

## OFF‐LABEL ANTIMICROBIAL DECLARATION

Authors declare no off‐label use of antimicrobials.

## INSTITUTIONAL ANIMAL CARE AND USE COMMITTEE (IACUC) OR OTHER APPROVAL DECLARATION

All samples were collected with informed client consent. This study was approved by the IACUC of National Taiwan University, Taipei, Taiwan (protocol code: IACUC No. NTU110‐EL‐00096).

## HUMAN ETHICS APPROVAL DECLARATION

Authors declare human ethics approval was not needed for this study.

## Supporting information


**Data S1:** Supporting Information.
