# Supplementary material for: Increased plasma DR‐70 (fibrinogen‐fibrin degradation products) concentrations as a diagnostic biomarker in dogs with neoplasms
Source: J Vet Intern Med. 2023 Oct 14;37(6):2391–401. doi: 10.1111/jvim.16898 (PMC10658483; doi:10.1111/jvim.16898)
Supplement: Supplementary file 1 — Data S1: Supporting Information. [file JVIM-37-2391-s001.pdf]

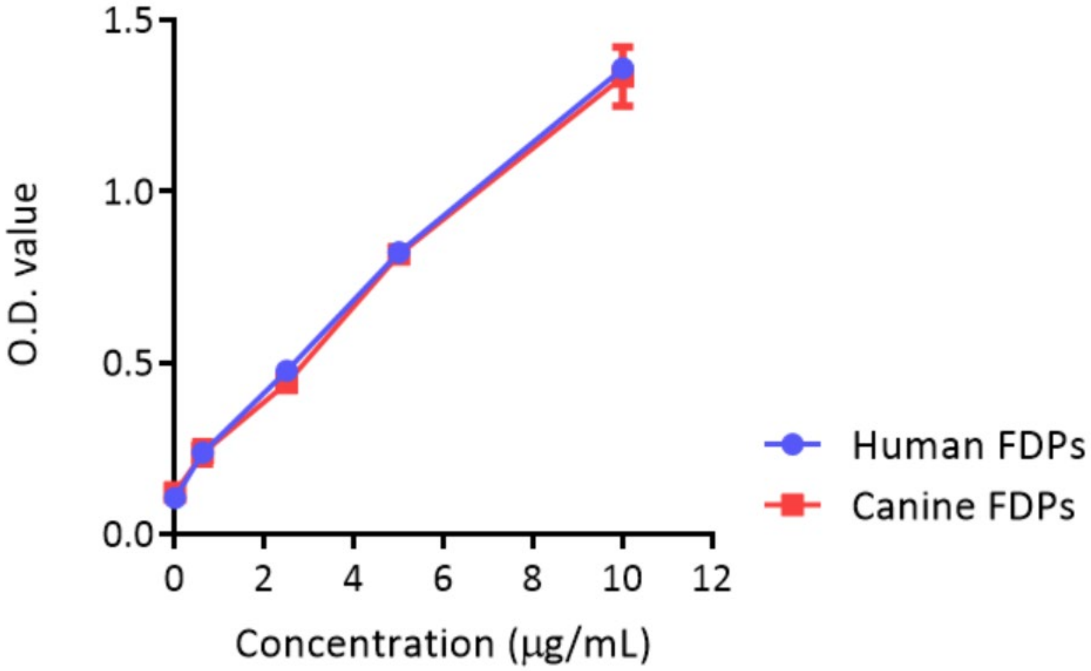

Figure S1. Comparison of human and canine FDPs in a commercial DR-70 ELISA kit. Data were described as mean ± SD.

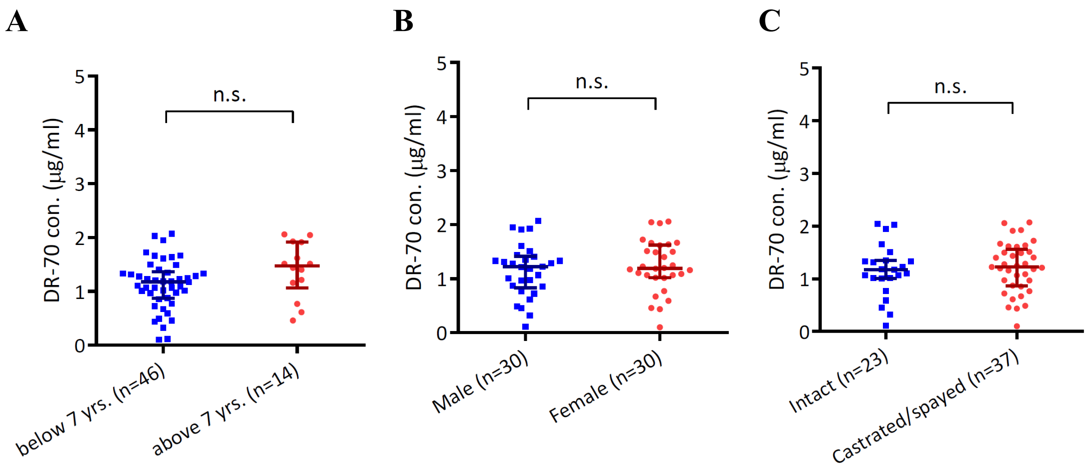

Figure S2. Comparisons of DR-70 concentrations in healthy dogs stratified by (**A**) age, (**B**) sex and (**C**) reproductive status. Data were described as median ± interquartile range and analyzed by the Mann-Whitney U test. *P* values <.05 were considered significant. n.s., no significant.

Table S1. Signalment of healthy dogs in the study.

	Breed	Sex	Age (years)	DR-70 (μg/mL)
1	Beagle	Male intact	3.5	1.313
2	Beagle	Male castrated	2.0	1.284
3	Beagle	Male castrated	4.0	0.851
4	Beagle	Female spayed	2.0	1.634
5	Beagle	Female spayed	3.8	1.490
6	Beagle	Female spayed	4.5	1.064
7	Border Collie	Male castrated	2.5	1.275
8	Border Collie	Male castrated	3.0	1.184
9	Border Collie	Male castrated	4.0	0.873
10	Border Collie	Female spayed	3.5	1.195
11	Chihuahua	Male intact	3.5	1.065
12	French Bulldog	Female spayed	6	0.670
13	French Bulldog	Female intact	4	1.010
14	Golden Retriever	Female spayed	2.0	1.666
15	Irish Setter	Male castrated	8.5	1.926
16	Irish Setter	Male intact	2.5	1.329
17	Irish Setter	Male castrated	2.5	1.225
18	Maltese	Male intact	4.6	1.009
19	Maltese	Male castrated	2.8	0.721
20	Maltese	Male castrated	5.5	0.968
21	Maltese	Male castrated	7.5	0.765
22	Miniature Dachshunds	Female intact	10.5	2.042
23	Miniature Dachshunds	Male intact	8	1.947
24	Miniature Dachshunds	Female intact	9	2.028
25	Miniature Dachshunds	Female spayed	9.5	1.618
26	Miniature Poodle	Male intact	10.5	1.509
27	Miniature Poodle	Male castrated	11	2.070
28	Miniature Poodle	Female spayed	9	2.059
29	Miniature Schnauzers	Female spayed	9	1.156
30	Miniature Schnauzers	Male castrated	8	1.911

31	Miniature Schnauzers	Male castrated	10.5	1.209
32	Mixed	Male castrated	2.5	0.973
33	Mixed	Male castrated	4.0	1.606
34	Mixed	Male castrated	8	0.612
35	Mixed	Male castrated	10	0.456
36	Mixed	Male castrated	9.5	1.434
37	Mixed	Female spayed	4.0	1.722
38	Mixed	Female spayed	6.0	0.100
39	Mixed	Female spayed	8.5	1.512
40	Mixed	Female spayed	9	1.400
41	Mountain Cur	Male intact	1.8	1.348
42	Mountain Cur	Male intact	2.6	1.329
43	Mountain Cur	Female intact	1.3	1.662
44	Mountain Cur	Female intact	1.3	1.017
45	Mountain Cur	Female intact	1.5	1.223
46	Mountain Cur	Female intact	3.0	1.103
47	Mountain Cur	Female intact	3.5	0.771
48	Mountain Cur	Female intact	3.5	1.063
49	Mountain Cur	Female intact	4.8	1.170
50	Mountain Cur	Female intact	5.5	0.456
51	Mountain Cur	Female spayed	6.0	0.435
52	Mountain Cur	Female spayed	6.0	1.498
53	Pembroke Welsh Corgi	Male intact	5.5	0.321
54	Pembroke Welsh Corgi	Female intact	4	0.588
55	Pembroke Welsh Corgi	Male castrated	3.8	0.488
56	Shiba Inu	Female intact	3	1.184
57	Shiba Inu	Male intact	6	0.112
58	Shih Tzu	Female spayed	3	1.246
59	Shih Tzu	Female spayed	4.6	1.087
60	Standard Poodle	Male castrated	2.5	1.405

Table S2. Description of the population of the tumor-bearing dogs

	Results
**Tumor types (numbers, n=263)**	
Adenocarcinoma	4
Carcinoma	1
Chondrosarcoma	2
Hepatic cell carcinoma	4
Leiomyosarcoma	1
Lipoma	1
Lymphoma	116
Mammary gland tumor	5
Mast cell tumor	17
Melanoma	12
Meningioma	2
Osteosarcoma	4
Perianal gland tumor	3
Plasmacytoma	2
Renal cell carcinoma	3
Sarcoma	9
Seminoma	1
Squamous cell carcinoma	4
Thyroid gland tumor	1
Transitional cell carcinoma	8
Trichoblastoma	2
Other	61
**Age (years, n=217)**	
Mean	8.9
Median	9
Mode	11
Minimum	1
Maximum	17

Table S3. DR-70 values in non-tumor dogs

No.	Diagnosis	Inflammatory status	Acute or chronic	DR-70 con. (μg/mL)
1	Chronic kidney disease	N	C	0.507
2	Chronic kidney disease	N	C	1.156
3	Congestive heart failure	N	C	1.222
4	Mitral insufficiency	N	C	1.510
5	Atopic dermatitis	I	C	1.611
6	Paralysis (IVDD)	N	C	1.639
7	Chronic hepatitis	I	C	1.653
8	Inflammatory bowel disease	I	C	1.681
9	Dislocation of hip	I	A	1.775
10	Inflammatory bowel disease	I	C	1.833
11	Perineum bleeding	N	C	1.911
12	Chronic dermatitis	I	C	1.926
13	Epilepsy	N	C	1.947
14	Trauma	I	A	1.986
15	Bone fracture	I	A	1.987
16	Bone fracture	I	A	2.028
17	Trauma	I	A	2.042
18	Acute pancreatitis	I	A	2.059
19	Trauma	I	A	2.070
20	Acute pancreatitis	I	A	2.188
21	Peritonitis	I	A	2.343
22	Poisoning (unknown)	N	A	2.372
23	Traffic accident	I	A	2.854
24	Poisoning (unknown)	N	A	2.942
